# How to interpret serum creatinine increases during decongestion

**DOI:** 10.3389/fcvm.2022.1098553

**Published:** 2023-01-04

**Authors:** Jonathan S. Chávez-Íñiguez, Juan B. Ivey-Miranda, Frida M. De la Vega-Mendez, Julian A. Borges-Vela

**Affiliations:** ^1^Nephrology Service, Hospital Civil de Guadalajara Fray Antonio Alcalde, Guadalajara, Mexico; ^2^University of Guadalajara Health Sciences Center, Guadalajara, Mexico; ^3^Heart Failure and Heart Transplant Clinic, Hospital de Cardiología, Instituto Mexicano del Seguro Social, Mexico City, Mexico

**Keywords:** AKI, cardiorenal syndrome 1, creatinine, decongestion, acute heart failure

## Abstract

During decongestion in acute decompensated heart failure (ADHF), it is common to observe elevations in serum creatinine (sCr) values due to vascular congestion, a mechanism that involves increased central venous pressure that has a negative impact on the nephron, promoting greater absorption of water and sodium, increased interstitial pressure in an encapsulated organ developing “renal tamponade” which is one of main physiopathological mechanism associated with impaired kidney function. For the treatment of this syndrome, it is recommended to use diuretics that generate a high urinary output and natriuresis to decongest the venous system, during this process the sCr values can rise, a phenomenon that may bother some cardiologist and nephrologist, since raise the suspicion of kidney damage that could worsen the prognosis of these patients. It is recommended that increases of up to 0.5 mg/dL from baseline are acceptable, but some patients have higher increases, and we believe that an arbitrary number would be impractical for everyone. These increases in sCr may be related to changes in glomerular hemodynamics and true hypovolemia associated with decongestion, but it is unlikely that they are due to structural injury or truly hypoperfusion and may even have a positive connotation if accompanied by an effective decongestion and be associated with a better prognosis in the medium to long term with fewer major cardiovascular and renal events. In this review, we give a comprehensive point of view on the interpretation of creatinine elevation during decongestion in patients with ADHF.

## Case presentation

A 66-year-old man was admitted to the emergency room with dyspnea and lower limb edema that worsened in the last 5 days. He has diabetes and hypertension in the past 14 years, chronic kidney disease G3aA2 (estimated glomerular filtration rate of 47 ml/min/1.73 m^2^ and albuminuria 122 mg) and heart failure with mildly reduced left ventricular systolic function. He last weight was 82 kg 1 month ago and now is 86 kg. He was taking losartan 50 mg every 12 h, dapagliflozin 10 mg, metformin 850 mg every 12 h, atorvastatin 20 mg and chlorthalidone 50 mg, the latter being stopped in the last 3 weeks because he forgot to take them on his short trip. His blood pressure was 176/96 mmHg, 92 beats per minute, 21 breaths per minute, oxygen saturation 93% at room air, jugular venous pressure at 20 cm H_2_O, lung ultrasound showed 16 B lines in 8 fields, a jugular vein radius < 2, VExUS score of 3, BNP 32,000 ng/dL, CA-125 88 mg/dL, and serum creatinine (sCr) 1.7 mg/dL (baseline 1.4 mg/dL 2 months ago). The patient received furosemide bolus followed by high-dose infusion, acetazolamide, continue the same previous doses of losartan, dapagliflozin and spironolactone for 3 days, seeking to achieve some of the results of the recent ADVOR and EMPULSE clinical trials, to promote greater natriuresis and urinary volume. At that time his urine output was 9.5 L, which resulted in improving dyspnea and lower limb edema, as well as a decrease in the VExUS score to 0. BNP decreased to 4,200 ng/dL, but sCr increased to 1.8, 2.5, and 2.9 mg/dL.

## Introduction

Cardiorenal syndrome is the most studied organic interaction in medicine. The type 1, is the one that combines the acute worsening of kidney function (WKF) after the acute worsening of cardiac function (1), often observed in patients with acute decompensated heart failure (ADHF) who develop acute elevation of sCr. It carries high morbidity and mortality, increasing up to 5 times the risk of dying within 28 days (2), being the most lethal of the five types of cardiorenal syndromes (3, 4). This is not surprising when observing the intricate pathophysiological mechanism that it possesses, but today it can be established that congestion is one of the main determinants of kidney disfunction, secondary to neurohormonal, inflammatory, and hemodynamic activation, which worsens the kidney inability to excrete sodium and water (5, 6), promoting compression of the kidney parenchyma, an entity recently described as “congestive nephropathy” (7).

## Impaired kidney function with ADHF

The worsening of the glomerular filtration rate (GFR) in congestive nephropathy could be explained by the following four mechanisms: (1) The kidney, being encapsulated, by increasing central venous pressure, promotes increased pressure in the interstitium, and inside the tubules, opposing the filtration force in Bowman’s capsule. (2) Enhanced activation of the renin angiotensin system (RAAS) and the sympathetic system increases interstitial oncotic pressure, which pulls water and Na+ from the tubule, leaving it without chloride and Na+. (3) The neurohormonal activation causes mesangial contraction, which decreases the surface area and capillary permeability, and (4) The hyperabsorption of Na+ increases the consumption of O_2_ in the renal tubule creating an hypoxic environment (8).

## Decongestion of type 1 cardiorenal syndrome with diuretics

The relief of severe decongestion should be considered an emergency and all possible therapeutic efforts should be made to improve this condition as soon as possible. In these cases, it is recommended to use diuretics and in refractory cases even extracorporeal ultrafiltration (9). It has been reported that the faster the decongestion, the better the prognosis. The Heart Failure Association of the European Society of Cardiology proposes how to administer loop diuretics with the therapeutic objective of achieving a urinary volume of >3–4 L per day (10) until reaching decongestion.

Furosemide is the most studied loop diuretic, it is suggested to administer large doses to achieve the goals, and the doses of intravenous furosemide used in different clinical trials vary greatly from that reported in the technical data sheet of the drug, doses ranging from 125 to 1,500 mg per day (11). In refractory cases, it has been suggested to apply sequential blockade of the renal tubule with different drugs that prevent the absorption of sodium and water in different sites, obtaining a response in up to 62% of the cases as evidenced by greater urinary volume (12). Options include adding drugs such as thiazides (13), mineralocorticoid receptor blockers (14), carbonic anhydrase inhibitors (15), sodium glucose transporter type 2 inhibitors (16, 17), and even hypertonic saline solutions (18). In a clinical trial carried out in patients with cardiorenal syndrome type 1, a sequential blockade strategy (with stable furosemide dose) was compared with increasing doses of furosemide. The trial reported similar effect in achieving recovery of kidney function, decongestion, and adverse events (19).

## Serum creatinine as a biomarker of kidney function in ADHF

Creatinine has great limitations when evaluating kidney function in patients with acute pathologies, where its values can change unpredictably and also take time to do so. Another great limitation is its generation because this is a complex process that depends on multiple metabolic steps that go from the interaction between arginine and glycine in the liver, pancreas and kidney, generating guanidinoacetate (glycocyamine), and then passing to the systemic circulation where it is metabolized to creatine and finally in the muscle is derived to phosphocreatine and creatinine (20). The latter is freely filtered and a small amount is secreted into the tubular lumen. During AKI the increase in sCr is due to a decrease in GFR and backleak through damaged proximal tubule cells (21), due to these complex steps, in different clinical scenarios, the increase in sCr does not always represent a true damage to the kidney parenchyma.

Another potential limitation of sCr is in states of fluid overload, commonly observed in AHF, where it might be possible Cr is affected by “dilution,” which interferes with its proper determination (22). That is one of the many reasons why the new biomarkers emerge with the intention to improve these limitations, improving the prediction of kidney injury, specifying the location of damage and ascertaining the severity and predicting the clinical evolution (23).

## Serum creatinine elevation during decongestion

Given the hypothesis of congestive nephropathy, diuretics should improve kidney function by reducing venous pressure (24). But this does not happen in all patients. During decongestion in patients with AHF, it has been reported that approximately 50% of patients increase sCr levels (25) or experience a 20% change in sCr (26–28). It has been suggested based on an expert’s opinion that sCr elevation < 0.5 mg/dL during decongestion of patients with AHF could be permissive (9), but many patients who achieve a successful decongestion exceed this value of sCr. It is in these cases where clinicians begin to doubt whether, despite achieving the objectives, they’re going in the right way. The classical paradigm would be to expect negative connotations with these sCr increases, but this phenomenon does not always represent an adverse event. A clear example that Cr elevations are associated with a better clinical course, such as a decrease in the risk of dying or presenting cardiovascular events is observed in the first 2 weeks of starting of sodium glucose type 2 inhibitors in diabetes-associated nephropathy (29), the start of RAASi (30), or in intense blood pressure control (< 120/80 mmHg) (31). This elevation of sCr induced with some treatments should be interpreted in the correct clinical context, which would be completely the opposite to the elevation that occurs in sepsis (32, 33), obstructive nephropathy (34), or post-surgery, where it is known extensively its pathological significance. Another example of this controversy occurs in some cardiac intensive care units, where it has been considered the initiation of continuous renal replacement therapies in patients with elevated sCr who meet the AKI KDIGO 2–3 criteria (35) without consider the full context.

Despite these weaknesses sCr has continued to be used, and in fact it has performed better than other biomarkers at predicting negative events in ADHF. For example, in a cohort of 787 patients with ADHF who were decongested several biomarkers were measured including brain natriuretic peptide, high sensitivity cardiac troponin I, galectin 3, serum neutrophil gelatinase-associated lipocalin, and urine neutrophil gelatinase-associated lipocalin. When considering elevation of sCr as the criterion for WRF, it was observed that no biomarker predicted WRF better than creatinine. Importantly, in the multivariable Cox analysis, brain natriuretic peptide, and high sensitivity cardiac troponin I, but not WRF, were significantly associated with the 1-year composite of death or heart failure hospitalization (36).

**FIGURE 1:**
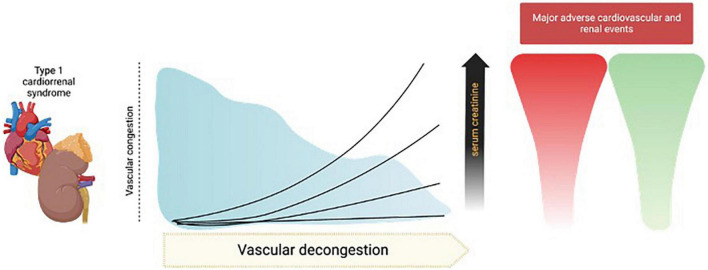
Causes of increase in serum creatinine (sCr) during decongestion. Hypotheses that are unlikely to play a critical role, such as ischemic damage that induces acute tubular necrosis and sCr hemoconcentration, are described in the panel **(left)**. In the panel **(right)**, the theories that are more likely and would have more prominence during the appearance of this event such as renal hypoperfusion, the induction of true intravascular hypovolemia and neurohormonal activation with hemodynamic alteration of the afferent and efferent glomerular arteries. The image was licensed from Biorender.

The reason why some patients increase sCr is not clear. Different pathogenic mechanisms have been proposed (37), as shown in Figure 1. Thus, in the following sections we will discuss potential causes of sCr elevation during decongestion in patients with ADHF, including those with greater or lesser biological plausibility and scientific evidence. Among those that are considered to have low quality evidence would be the hypotheses of intense parenchymal injury that would have consequently acute tubular necrosis. True tubular parenchymal damage during this scenario is an unusual event. A *post hoc* analysis of the clinical trial CARRESS-HF evaluated other biomarkers in addition to sCr during decongestion. The authors described that effective decongestion was associated with increased urinary renal tubular injury biomarkers such as N-acetyl-glucosaminidase (NAG), kidney injury molecule-1 (KIM-1), and neutrophil gelatinase-associated lipocalin (NGAL), and importantly, they reported that WKF increased up to 12 times the possibility that these tubular biomarkers increase. However, increases in urinary renal tubular biomarkers were paradoxically associated with better kidney recovery at 60 days (25) (Figure 1A).

Another explanation for the elevation of sCr with scare clinical evidence is hemoconcentration that happens during diuretics in ADHF patients. Historically, it has been postulated that during intense decongestion, the increase in sCr may be due to an effect of hemoconcentration, but this was demystified in a *post hoc* study of the ROSE clinical trial. In that study 270 patients with ADHF and aggressive diuresis during 3 days were analyzed, and it was observed that the change in sCr may be due to hemoconcentration, only in those patients with urinary output > 7.5 L, an unusual event with the usual management, emphasizing that the hemoconcentration of sCr would not be the cause in most of these cases (38) (Figure 1A). On the other hand, the most plausible explanations would be alterations in renal perfusion, true intravascular hypovolemia, and neurohormonal activation with hemodynamic changes in the glomerular arteries.

This elevation of sCr could represent a state of relative hypovolemia, in which decreasing left ventricular end-diastolic pressure decreases renal perfusion to a pathological point. Loop diuretics can profoundly worsen tubuloglomerular feedback because they inhibit the sodium/potassium/2-chloride cotransporter, which promotes large amounts of these electrolytes to travel through the tubule, the macula densa sense chloride, then adenosine release falls, leading to afferent arteriolar vasodilation, and increased renin release from granular cells. Neurohormonal activation causes efferent and, to a lesser extent, afferent arteriolar vasoconstriction, causing decreased GFR due to hypoperfusion (39, 40) (Figure 1B).

Due to the changes and interactions of various markers and metrics during decongestion, it is relevant to consider the usefulness of guiding decongestion using a “multiparametric view” beyond sCr elevation objectively determining intravascular volume and assess bedside systolic volume with FoCUS as an indicator of intravascular hypovolemia.

## Clinical significance of decongestion and creatinine increases

### Bad creatinine elevation

Previous studies have shown that WKF during decongestion in ADHF has negative connotations, including very small increases in sCr (> 0.1 mg/dL) that would have a negative impact (41). In a study of 1,004 patients with ADHF, increased sCr > 0.3 mg/dL occurred in 1 out of 5 patients and was associated with prolonged hospitalization, and mortality (42). A meta-analysis of eight studies and 18,634 patients with HF, the authors defined WRF as increased sCr > 0.2 mg/dL or impaired GFR > 5 ml/min/1.73 m^2^, they reported that this event occurs in 25% of patients and was associated with increased 62% the risk of dead and had a stronger association with higher increases in sCr (43). An important limitation of this study was that they did not consider whether these patients reached decongestion during this process. Another meta-analysis of 28 studies and 49,890 patients reported that WRF occurs in 23% of cases and was associated with a 95% increased risk of death (44), but again, it does not analyze whether decongestion was achieved.

It is noteworthy that a study of 52 patients with type 1 cardiorenal syndrome refractory to diuretics where they were treated with kidney replacement therapy (KRT), the sCr values were not different between the groups of patients who recovered kidney function after follow-up, those who were dependent on KRT or those who died (45), a result that could be interpreted in favor of the fact that the severity of WKF is not assessable only with sCr levels.

But contemporary data have consistently indicated that WKF is not always associated with poor outcomes. And they have changed the notion that sCr elevations are always due to kidney pathological changes, it is alternatively proposed that hemodynamic and functional changes of the glomerulus may occur (46). It is important to always consider other etiologies of elevation of sCr, especially when this increase is out of the ordinary, such as in the presence of sepsis, bleeding, or nephrotoxic drugs.

Since the elevation of sCr in this context may raise doubts, attempts have been made to carry out these evaluations parametrically, together with other markers of decongestion, such as urinary sodium, NT-proBNP, BNP, CA-125 or the diuretic response to loop diuretics. The trajectory of some biomarkers that suggest decongestion in AHF has been considered, such as the decrease in BNP or NT-proBNP of at least 30%, body weight loss of 2.5 kg and decrease of CA-125. Likewise, hemoconcentration as assessed by increases in hemoglobin, hematocrit, serum albumin or total protein is accepted as a marker of decongestion (47).

## Better prognosis with sCr elevations in decongestion

In this section, we discuss the evidence that has shown that the elevation of sCr in those who reach the goals of decongestion is associated with a better prognosis.

One of the first attempts to clarify this question was a study that evaluated the worsening of GFR according to decongestion parameters, such as increased hematocrit, total protein, or serum albumin. It was observed that decongested patients who increased these parameters were associated with a 69% less probability of dying, in comparison to those who did not (48).

In a *post hoc* of the ROSE study that evaluated changes in sCr and cystatin C in 238 patients with ADHF, decongestion was achieved with high doses of furosemide, reaching up to 8,726 ml in 3 consecutive days. It was observed that those patients who had an increase in both biomarkers were the group with the best survival after a follow-up of more than 150 days, reducing the probability of dead by 20% (49).

In another retrospective cohort of 4,182 patients with ADHF treated with diuretics, it was observed that 21% of them had elevated sCr and hematocrit at hospital discharge. Those patients had worse GFR, more days of hospitalization, higher dose of diuretics, but higher urine volume, greater fluid loss and weight loss. Notably, this translated to a 20% decrease in the probability of dying in a long follow-up of more than 3 years (50).

It has been reported in six cohorts and 1,232 hospitalized patients with AHF that a decrease in NTproBNP of > 30% during hospitalization is associated with a lower probability of dying, despite WKF (51).

The *post hoc* analysis of the AKINESIS study showed that the rapid decrease in BNP of >30% on the first day of decongestion in ADHF was associated with being free of congestion events in long follow-ups, this benefit was also accompanied by increases in sCr, and again, this WKF was irrelevant in the survival analysis (52).

In a *post hoc* of the DOSE study, they describe how the probability that the event composed of death, rehospitalization or visits to the emergency services 60 days after hospital discharge of patients with ADHF is increased in those patients who, during decongestion with diuretics, paradoxically have a decrease in sCr, an event that was not observed when it was increased (53).

The *post hoc* analysis of the EVEREST clinical trial, in 3,500 patients, showed that markers of aggressive decongestion that improved when treated with diuretics in ADHF, such as decreased values of BNP, NT-proBNP, congestion scores or increments of hematocrit, albumin and total proteins are associated with lower probabilities of being diagnosed with CKD grade 4 after a long follow-up of 10 months, reducing this risk by 70% (54).

In two cohorts (POROTEC and RELAX-AHF-2) the increases of sCr (> 0.3 mg/dL on day 4 of hospitalization) was analyzed according to the response to diuretics (defined as the reduction of 350 g of weight for every 40 mg of furosemide) and it was observed that the best survival at 180 days was in those who responded to diuretics, regardless of whether they presented WKF or not (55).

Therefore, the parameters that we currently use to describe decongestion in ADHF must be interpreted together and never alone. Therefore, the parameters we currently use to describe decongestion in ADHF should be interpreted together. A clear example of this suggestion is reported in the *post hoc* analysis of the EVEREST study, the authors described in 3,715 patients followed for 10 months, that the greatest changes in kidney function parameters lose negative prognostic value when analyzed together with the largest changes in BNP and hematocrit (54).

There is even a meta-analysis of 13 cohorts and 8,238 patients with type 1 cardiorenal syndrome in which the authors report that, if effective decongestion is not achieved, the appearance of AKI is associated with a > twofold increase in the risk of death, an effect that is nullified if decongestion was achieved (56).

Chirag R. Parikh and Steven G. Coca have proposed that these increases in sCr in decongestion should not be called AKI, at least not as the one described by KDIGO with its negative associations, instead terms such as “permissive hypercreatininemia” or “Permissive AKI” (24) and thereby change the landscape and traditional conceptualization.

## How much should be acceptable in the magnitude of the sCr increase in AHF decongestion?

**FIGURE 2:**
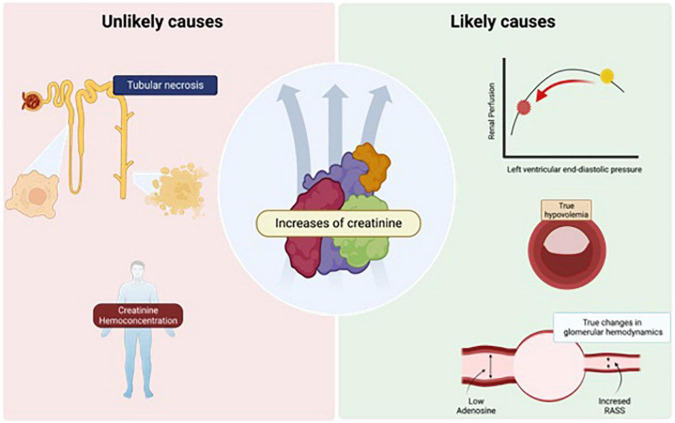
Different trajectories of serum creatinine (sCr) during decongestion in cardiorenal syndrome 1. During effective decongestion in acute decompensated heart failure (ADHF), the increase in sCr < 0.5 mg/dL has been considered acceptable, and is associated with fewer major cardiorenal events, this arbitrary number seems to be inconsistent with reality, where even greater increases in sCr could be associated with the same benefit, but there could be patients whose values have negative connotations and reflect a worsening of kidney function that exceeds the benefit of decongestion. The image was licensed from Biorender.

For decades, increases in sCr during decongestion have had many names, such as WKF or AKI, but authors have also been referred to solely as the numerical increase in sCr or as a percentage, increases in sCr > 0.3 mg/dL, > 20%, > 25%, or even > 50% have been proposed (56) (Table 1). We believe that a specific number has not yet been established with robust and convincing evidence and should not be systematically adopted for clinical decision making. There are patients who experience greater increases in sCr and still continue to have a favorable evolution, as during follow-up they recover their kidney function. However, we also consider that there are unacceptable values (for example, > 7.0 or 12 mg/dL). We believe that there could be an individual “sweet spot,” according to the clinical characteristics of each patient and the conditions established during management. Notwithstanding, for now, unfortunately, it continues to be an artisan response that involves clinical experience, profound physiological knowledge of intravascular volume, hemodynamics, diuretics, and decongestion (Figure 2).

**TABLE 1:** Definitions of worsening kidney function, its frequency, and main outcomes during decongestion in acute decompensated heart failure.

References	Definition of WKF	% WKF	Conclusion
Breidthardt et al. (57)	Increase sCr ≥ 0.3 mg/dL	33.2	Decongestion assessed by hemoconcentration was associated with lower mortality in patient with or without WKF.
Brisco et al. (53)	Increase sCr ≥ 0.3 mg/dL	15.5	Changes in serum creatinine should not be used as a surrogate endpoint in trials of decongestive strategies.
Fudim et al. (58)	Increase sCr ≥ 0.3 mg/dL	44.8	In-hospital WKF was not associated with increased hazard of all-cause mortality among patients successfully decongested at discharge.
Martins et al. (59)	Increase sCr ≥ 0.3 mg/dL	49	WKF with hemoconcentration is associated with a better prognosis, similar to that of patients without WKF.
Metra et al. (60)	Increase sCr ≥ 0.3 mg/dL	50.2	WKF alone is not an independent determinant of outcomes in patients with AHF. It has an additive prognostic value when it occurs in patients with persistent signs of congestion.
Metra et al. (61)	Increase sCr ≥ 0.3 mg/dL	20.5	WKF was associated with longer length of hospital stay, and worse 30- and 90-day outcomes. However, effects were largely driven by patients who had residual congestion at the time of renal function assessment.
Rao et al. (25)	Increase sCr ≥ 0.3 mg/dL	26.4	The benefits of decongestion may outweigh any modest or transient increases in serum creatinine or tubular injury markers that occur during intensive volume removal.
Salah et al. (51)	Increase sCr ≥ 0.3 mg/dL and 25%	10.9	In ADHF patients it may be warranted to strive for an optimal decrease in NT-proBNP, even if this induces WKF.
Sokolski et al. (62)	Increase sCr ≥ 0.3 mg/dL or eGFR decrease > 25%	14.3	4% of patients have true WKF defined as a rise in creatinine levels and/or a drop in eGFR in combination with an unfavorable clinical course.
Stolfo et al. (63)	≥ 20% decrease in eGFR	23	WRF does not affect the prognosis of ADHF and, when associated with a significant BNP reduction, identifies patients with adequate decongestion at discharge and favorable outcome.
Testani et al. (64)	≥ 20% decrease in eGFR	20.5	However, WKF that occurs in the setting of SBP-reduction is not associated with an adverse prognosis, whereas WKF in the absence of this provocation is strongly associated with increased mortality.
Wattad et al. (65)	Increase sCr ≥ 0.3 mg/dL	27	There is a substantial interaction between persistent congestion and WKF such that congestion portends increased mortality particularly when associated with WKF.
Wettersten et al. (66)	Increase sCr ≥ 0.3 mg/dL or > 50%	32.2	Decreased BNP was associated with better in-hospital and long-term outcomes. WRF was only associated with adverse outcomes in patients without decreased BNP.

ADHF, acute decompensated heart failure; BNP, brain natriuretic peptide; eGFR, estimated glomerular filtration rate; sCr, serum creatinine; SBP, systolic blood pressure; WKF, worsening kidney function.

## Back to the case

After 6 days of decongestion with diuretics, the dyspnea disappeared, BNP decreased to 2,100 ng/dL, the patient had a total urinary volume of 11 L, and the sCr value decreased to 1.6 mg/dL. Upon discharge, patient was prescribed with losartan 50 mg every 12 h, dapagliflozin 10 mg, metformin 850 mg, atorvastatin 20 mg, and chlorthalidone 50 mg, with strict instructions to not discontinue them. After 2 months of his hospitalization, he refers a normal life, with routine physical activity and without dyspnea or edema, blood pressure is 120/78 mmHg, sCr had returned to its baseline value 1.4 mg/dL and serum sodium is 136 mEq/L, potassium 4.1 mEq/L, glucose 122 mg/dL, and CA-125 is 11 mg/dL.

## Author contributions

JC-Í, JI-M, FD, and JB-V contributed equally to the realization of the manuscript. All authors contributed to the article and approved the submitted version.
